# Health Transformation Plan for Universal Health Coverage in Iran: Reflections From the Past

**DOI:** 10.34172/ijhpm.8817

**Published:** 2025-04-16

**Authors:** Sameen Siddiqi

**Affiliations:** Department of Community Health Sciences, Aga Khan University, Karachi, Pakistan.

 Serving in the Islamic Republic of Iran as the Head of the World Health Organization’s (WHO’s) Country Office in Tehran is considered a privilege by WHO’s international staff. When invited to serve in this position in 2016, I did not hesitate the least since such an experience is as much a learning opportunity as it is to advise the country’s dedicated leadership. During that period, some of the developments in health and the experience of living in Iran have left an indelible mark that distinguishes this country of almost ninety million people.

 The primary healthcare (PHC) system of Iran is well recognized globally for its pivotal role since the 1980s in terms of providing accessible and equitable services to its rural population through a network of PHC facilities reinforced by the presence of the well-known *Behvarz* or the community health workers, who have played a cardinal role in influencing health outcomes in the country.^1,2^ During the period of my service, more intriguing was to observe the well-established urban PHC services given that over 70% of the Iranian population was living in the cities by then.^3^ Despite resource limitations, the extensive network of urban PHC facilities offered an elaborate skill mix of staff that included a psychologist, nutritionist, and an environmental health expert meant to respond to the high burden of mental health problems, obesity and noncommunicable diseases. While some cadres such as psychologists required more training, that was more a matter of time. Many low- and middle-income countries that struggle to establish a system of urban PHC^4^ can benefit from the Iranian experience.

 Indeed, the health system of Iran has relied heavily on the distinctive approach of integrating healthcare delivery with medical education. Not without its critics, this arrangement gives immense authority and responsibility to the chancellors of over 60 medical universities across the country not only to offer medical and allied educational programs but also to deliver the entire range of health services from primary to tertiary. The chancellors report directly to the minister of health who is supported by several deputies. Despite efforts to separate health from medical education, the system remains well-established and has been functioning for over four decades.

 A corollary to the integration of healthcare and medical education in Iran is the unique feature of the ministry of health, led and managed predominantly by academic health professionals, which has its pros and cons. Among the pros was the continued discussion, intellectual debate, and a freedom to express views in forums that is not often seen in ministries of health. This was not without its cons as academics are often not the best managers. Additionally, ministerial staff had frequently to return to their universities as professors once they ran out of favor of the policy-makers.

 The principal reason for being invited to serve as the Representative of WHO to Iran was my prior experience of supporting health system reforms in several countries, and Iran was in the middle of an ambitious Health Transformation Plan (HTP)^5^ with the aim of achieving universal health coverage (UHC) despite the international sanctions imposed by the western powers. Inspired by HTP of Turkey and led by the then Minister of Health, the HTP was a major health reform being implemented by the government of Iran in 2014. The main objective of HTP was to improve the quality and accessibility of healthcare services, especially for vulnerable populations and reduce the level of out-of-pocket spending on health.^6^ For this purpose, huge amounts of additional financial resources were mobilized by earmarking a proportion of the value added tax and revenue from oil for health, amounting to several billion US dollars. In addition to expanding coverage to include the entire population by bringing them within the fold of the different social health insurance schemes, HTP included elaborate plans to build new hospitals and health centers, as well as to upgrade existing facilities with modern equipment, infrastructure, and technologies.

 The commitment of the government of Iran could not be underestimated as the total expenditure on health was almost US 350 per capita, which was much more than Thailand, the latter being the first among the low- and middle-income countries to achieve UHC. There were some opportunities missed for improvement within HTP, for instance, an essential package of health services^7^ accessible to all could not be delineated leading to frivolous use of services. The tariffs for healthcare interventions were thought to favor specialists that put a question mark on HTPs sustainability. The health insurance organizations were functioning under the Ministry of Social Affairs and did not necessarily align with the policies of the Ministry of Health and Medical Education or encouraged merger to a single payer system as was the case in Turkey. Also, there was the problem of duality of insurance, and a sizeable proportion of the population was insured under more than one scheme.

 Despite these challenges, by 2017 HTP managed to cover more than 90% of the population, large number of healthcare institutions were refurbished and upgraded, and there was reduction in share of out-of-pocket spending from over 50% to around 40% of the current health expenditure but could not reach the UHC benchmark of less than 20%. All these achievements were by no means small for a country that has been under international sanctions for decades and was a true reflection of the commitment of its leadership to achieve UHC.

 Finally, there was also a life in Iran beyond work at the WHO, which should not be missed. Iranians are one of the most hospitable societies, always welcoming and willing to help. The rich history and culture of the country from Isfahan to Kerman and from Shiraz to Mazandaran are a source of great learning and enhancement for its visitors. Indeed, no stay in Iran would be complete without a visit to the tomb of *Ibn Sina* the preeminent philosopher and physician of the Muslim World in the beautiful province of Hamadan!

## Ethical issues

 Not applicable.

## Conflicts of interest

 Author declares that he has no conflicts of interest.
